# Effect of arm sling application on gait and balance in patients with post-stroke hemiplegia: a systematic review and meta-analysis

**DOI:** 10.1038/s41598-021-90602-y

**Published:** 2021-05-27

**Authors:** Lien-Chieh Lin, Chun-De Liao, Chin-Wen Wu, Shih-Wei Huang, Jia-Pei Hong, Hung-Chou Chen

**Affiliations:** 1 Department of Physical Medicine and Rehabilitation, Shuang Ho Hospital, Taipei Medical University, No. 291 Zhongzheng Road, Zhonghe District, New Taipei City, 235 Taiwan; 2 Master Program in Long-Term Care, College of Nursing, Taipei Medical University, Taipei, Taiwan; 3 Department of Physical Medicine and Rehabilitation, School of Medicine, College of Medicine, Taipei Medical University, Taipei, Taiwan; 4 Center for Evidence-Based Health Care, Shuang Ho Hospital, Taipei Medical University, New Taipei City, Taiwan

**Keywords:** Health care, Medical research, Neurology

## Abstract

Hemiplegic shoulder pain and impairment are common poststroke outcomes, for which arm slings constitute long-used treatments. Although multiple studies have suggested association between gait pattern and sling application, results have varied. Accordingly, we conducted this meta-analysis to determine how arm sling use affects the gait and balance of patients with poststroke hemiplegia. The PubMed, Embase, and Cochrane Library databases were searched until April 21, 2021, for randomized or quasi-randomized controlled trials evaluating the effect of arm slings on gait or balance in patients with poststroke hemiplegia. The primary outcome was walking speed; the secondary outcomes were functional balance tests or walking evaluation parameters for which sufficient analytical data were available in three or more studies. Nine studies with a total of 235 patients were included, all of which were within-patient comparisons. Six studies reported significant between-group differences in walking speed with and without the use of arm slings. Patients wearing arm slings had higher walking speed (standardized mean difference =  − 0.31, 95% confidence interval [CI] =  − 0.55 to − 0.07, *P* = 0.01, n = 159; weighted mean difference =  − 0.06, 95% CI − 0.10 to − 0.02, *P* = 0.001, n = 159). Our findings suggest that arm sling use improves gait performance, particularly walking speed, in patients with poststroke hemiplegia.

## Introduction

Shoulder pain with subluxation was a common complication in patients with post-stroke hemiplegia^1^. In those with substantial upper limb weakness, vertical glenohumeral subluxation can develop, causing a downward migration of the humerus, eventually resulting in hemiplegic shoulder pain^2, 3^. One study observed that 17% and 23% of stroke survivors reported experiencing certain symptoms 1 week and 6 months poststroke, respectively. Moreover, even those with no or mild sensorimotor deficit might develop shoulder pain^4^. Other investigations have further demonstrated correlations between upper extremity hemiplegia and gait speed, for which various theories have been proposed. For example, Bovonsunthoncha et al. and Brooke et al. have indicated that reduced and unsupported arm movement on the affected side may negatively affect ankle range of motion^5, 6^. In addition, Hesse et al. indicated that asymmetrical gait patterns and a sense of insecurity caused by upper limb impairment were possibly correlated with abnormal gait patterns^7^.

Assistive support systems such as arm slings have long been used to treat shoulder subluxation after stroke^1, 8^. One systemic review reported improvement of shoulder pain and subluxation when applying arm sling among individuals with stroke^2^. As for association between gait and arm sling, Södring et al.’s 1980 study was one of the earliest to investigate the effect of arm sling use on posture and gait peformance. The patients reported better standing and walking balance while using arm sling^9^. Although trials have evaluated the effect of arm sling use on gait or balance of patients with post-stroke hemiplegia in the past two decades, the result of their findings have varied^7, 10–17^. Therefore, we conducted this study, which would be the first systematic review with meta-analysis, to determine how arm sling use affects the gait and balance of patients with post-stroke hemiplegia.

## Methods

### Searching strategy and data sources

The PubMed, Embase, and Cochrane Library databases were searched for relevant studies, with the final search performed on April 21, 2021. No language or geographic restrictions were applied; furthermore, no filters were used, and any article tags or labels were disregarded. The following terms, words, and combinations of words were used in the systematic search in all databases: (arm OR shoulder OR (upper limb*)) AND (sling* OR brace* OR orthosis OR orthoses) AND (balance OR gait OR walk* OR posture OR ambulat* OR ataxia). All included studies were also entered into the “similar articles” function and the science citation index in the PubMed database. We identified additional studies by manually searching the reference sections of these papers and by contacting known experts in the field.

### Eligibility criteria

Eligibility criteria for study inclusion were as follows: (1) randomized or quasi-randomized controlled trials evaluating the effect of arm sling for gait or balance in patients with post-stroke hemiplegia that (2) clearly described the patient inclusion and exclusion criteria and (3) adequately described the intervention and placebo. Study location, patient age, and journal type were not limited. Studies were excluded for one or more of the following criteria: (1) different target populations, (2) endpoints unrelated to gait or balance, or (3) overlapping with other interventions.

### Data extraction and quality assessment

Two reviewers independently reviewed the full texts of relevant articles to identify publications that fulfilled the inclusion criteria. They then perform the data extraction, including data on the participants, inclusion and exclusion criteria, intervention details, and outcome measurement. The two reviewers’ results were compared, and any discrepancies or disagreements were resolved through discussion with a third reviewer, who evaluated the same data. The authors of the studies were contacted for additional information when necessary. The risk of bias in the included trials was assessed through the Physiotherapy Evidence Database (PEDro) scale, which is widely used to assess the risk of bias in randomized controlled trials^16^.

### Outcome selection, data synthesis and analysis

Walking speed was the primary outcome used to evaluate the effect of arm sling use on gait and balance. The secondary outcomes were functional balance tests or other parameters evaluating walking for which sufficient analytical data were available in three or more studies. If a study had more than one functional balance outcome, we selected the one most commonly used in other studies. Statistical analyses were performed using the statistical package Review Manager, version 5.4 (Cochrane Collaboration, Oxford, England; https://training.cochrane.org/online-learning/core-software-cochrane-reviews/revman/revman-5-download). We used the mean change from baseline of each treatment arm to represent the outcome changes. The standardized mean differences (SMD) with 95% confidence intervals (CI) were calculated from the meta-analysis data^18^. The weighted mean differences (WMD) of primary outcome was also provided. Data were pooled using the random effects model to account for the variation in the study methods. Statistical heterogeneity was calculated using the *I*^2^ test.

## Results

**Figure 1:**
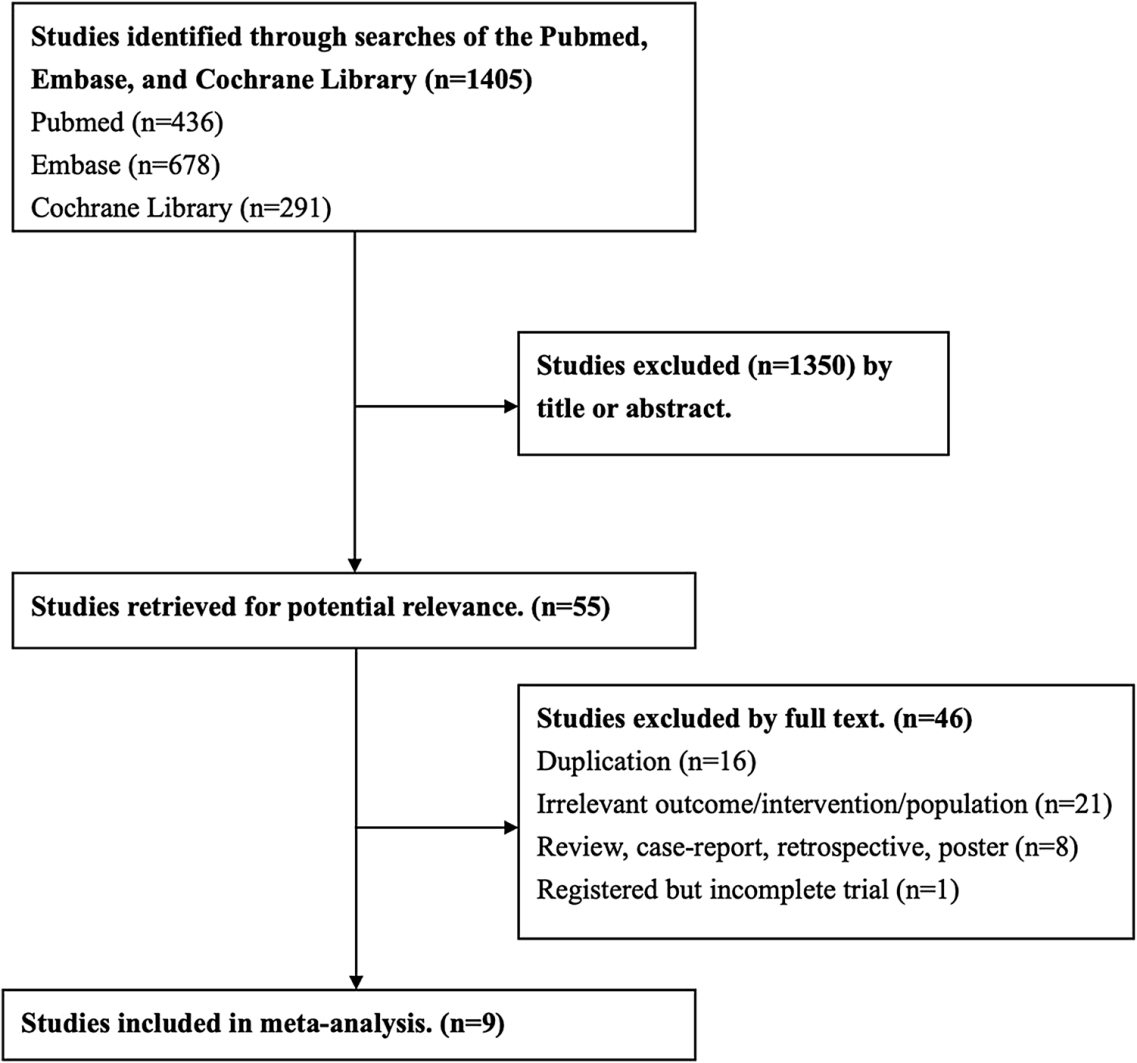
Flowchart of trial selection.

The review process is outlined in Fig. 1. The initial search yielded 1405 studies, 1350 of which were deemed ineligible through screening of titles and abstracts. Subsequently, the full text of 55 studies was screened in great detail for potentially applicable data. Of these, 46 did not meet the eligibility criteria. Nine eligible trials were included in the present study^7, 10–17^.

The main characteristics of the studies are listed in Table 1. The publication dates were between 2002 and 2018 and the sample sizes ranged from 9 to 57. All nine studies had crossover designs, but three was not randomized. Four studies provided a specific model of arm sling^7, 10, 12, 17^. The others described the orthosis in more simple terms, including “arm sling,” “elastic arm sling,” or “simple arm sling.”

**Table 1:** Characteristics of included trials.

Study	Inclusion criteria	Study design	Intervention	Wash-out period	Age / Numbers	Included outcomes
Jeong et al., 2017; Korea^17^	1. First stroke, hemiplegia; glenohumeral subluxation 2. Ambulation with cane; FAC score 4–5 3. MMSE score > 23	Crossover; randomized	Vest-type shoulder forearm support	1 h	Single*: 59.57 ± 8.57 / 30	Walking speed
					Quad*: 56.85 ± 11.73 / 27	
Sohn et al., 2015; Korea^16^	1. Hemiplegic stroke 2. Stand > 30 s w/o assistive devices 3. Follow commands	Crossover; randomized	Simple arm sling	40 min	57 ± 12 / 27	Berg Balance Scale
Hwang et al., 2015; Korea^15^	1. Stroke > 6 months prior 2. FAC score > 3 3. Follow simple commands	Crossover; unclear allocation	Elastic arm sling	5 min	61.18 ± 10.79 / 13	Walking speed Stride length Cadence
Han et al., 2011; Korea^10^	1. First stroke by CT or MRI 2. Fo llow commands and walk independently 3. Brunnstrom stage < 4 of the upper extremity	Crossover; randomized	Vest-type shoulder forearm support	20 min	61.3 ± 9.3 / 37	Walking speed
Acar and Karatas, 2010; Turkey^14^	1. Diagnosis of stroke	Crossover; alternate allocation	Arm sling	1 day	59.3 ± 16.8 / 26	Berg Balance Scale
Yavuzer and Ergin, 2002; Turkey^13^	1. First stroke and hemiplegia 2. Follow commands and walk independently	Crossover; randomized	Arm sling	Unknown	53.1 ± 9.7 / 31	Walking speed Step length
Anke et al., 2018; Belgium^12^	1. First stroke within 9 months 2. Stand w/o assistive devices > 3 min; walk > 20 m 3. Follow commands	Crossover; randomized	Shoulderlift Actimove	Unknown	50.67 ± 7.11 / 9	Walking speed Stride length Cadence
Şahin et al., 2012; Turkey^11^	1. First stroke and hemiplegia 2. Stand independently for 2 min	Crossover; randomized	Arm sling	30 s	53.91 ± 11.34 / 23	Kinesthetic Ability Trainer 3000
Hesse et al., 2013; Germany^7^	1. First supratentorial stroke 2. Nonfunctional upper extremity (shoulder subluxation) 3. Walk > 20 m and undergo a short interview	Crossover; unclear allocation	Shoulder orthosis	10 min	Unknown / 12	Walking speed Stride length Cadence

FAC, Functional Ambulation Category; MMSE, Mini Mental State Examination; CT, computed tomography; MRI, magnetic resonance imaging; w/o, without; Single*, Single-cane users; Quad*, quad-cane users; min, minutes; m, meter.

Table 2 presents the details of the nine included studies, whose quality we assessed using the PEDro scale. All studies were of medium overall quality. Due to arm sling’s characteristics as an intervention, concealed allocation, subject blinding, therapist blinding, and assessor blinding were not achievable. Similarity at baseline was easily achieved because all the studies had crossover designs. However, random allocation was not described in three studies^7, 14, 15^. For those who had performed random allocation, the randomization was mainly achieved by wearing arm sling and not wearing arm sling in randomized orders. Hesse et al. recruited 40 patients from two facilities, and they performed comparison trial for patients from one of the two units^7^.

**Table 2:** Summary of the physiotherapy evidence database (PEDro) scale.

	1	2	3	4	5	6	7	8	9	10	Overall quality*
Jeong et al., 2017^17^	✓		✓				✓	✓	✓	✓	Medium
Sohn et al., 2015^16^	✓		✓				✓	✓	✓	✓	Medium
Hwang et al., 2015^15^			✓				✓	✓	✓	✓	Medium
Han et al., 2011^10^	✓		✓				✓	✓	✓	✓	Medium
Acar and Karatas, 2010^14^			✓				✓	✓	✓	✓	Medium
Yavuzer and Ergin, 2002^13^	✓		✓				✓	✓	✓	✓	Medium
Anke et al., 2018^12^	✓		✓				✓	✓	✓	✓	Medium
Şahin et al., 2012^11^	✓		✓						✓	✓	Medium
Hesse et al., 2013^7^			✓				✓	✓	✓	✓	Medium

1, random allocation; 2, concealed allocation; 3, baseline similarity; 4, subject blinding; 5, therapist blinding; 6, assessor blinding; 7, more than 85% follow-up for at least one key outcome; 8, intention-to-treat analysis; 9, between-group statistical comparison for at least one key outcome; 10, point and variability measures for at least one key outcome.

*Methodological quality: high, ≥ 7 points; medium, 4–6 points; low, ≤ 3 points.

w/o: without.

### Walking speed

**Figure 2:**
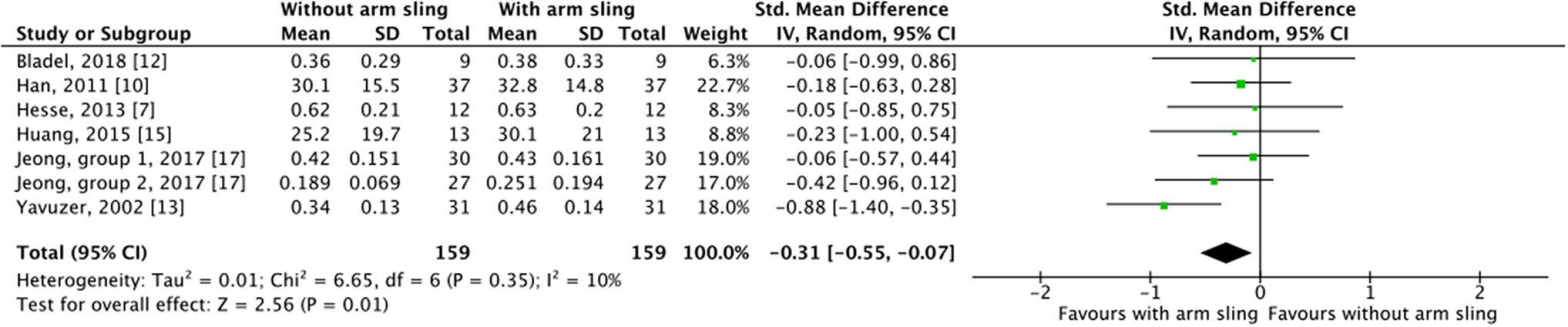
Forest plot for walking speed comparison, standard mean difference.

**Figure 3:**
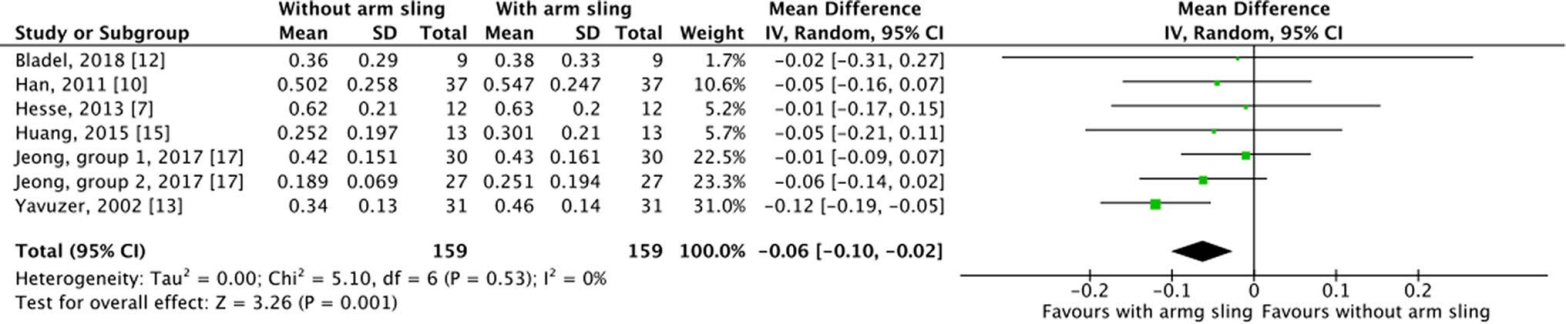
Forest plot for walking speed comparison, weighted mean difference.

Six studies examined the walking speed of patients using and not using an arm sling^7, 10, 12, 13, 15, 17^. The patients examined in Jeong et al. were divided into two groups, one using single canes and the other using quad canes. We included both in our analysis. The patients in the other five studies did not use walking aids. As Figs. 2 and 3 demonstrates, significant between-group differences were observed in both SMD (*P* = 0.01) and WMD (*P* = 0.001). When using an arm sling, patients with hemiplegia had higher walking speed (SMD =  − 0.31, 95% CI − 0.55 to − 0.07, n = 159; WMD =  − 0.06, 95% CI: − 0.10 to − 0.02, n = 159). The results indicated low heterogeneity among the studies (*I*^2^ = 10% and *I*^2^ = 0% for SMD and WMD, respectively).

### Balance tests

**Figure 4:**
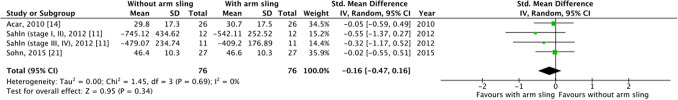
Forest plot for functional balance tests comparison.

Three studies examined the balance performance of the patients using and not using an arm sling^11, 14, 16^. The patients Şahin et al. examined were divided into two groups according to their Brunnstrom stage. We included both in our analysis. The assessment method used was the Kinesthetic Ability Trainer 3000. Sohn et al. and Acar and Karatas both used multiple assessment methods, including the Berg Balance Scale. As Fig. 4 shows, no significant between-group differences were observed (*P* = 0.69). However, an evident trend favoring the balance performance of the arm sling-using group was noted (SMD =  − 0.16, 95% CI − 0.47–0.16, n = 76). The results indicated low heterogeneity among the studies (*I*^2^ = 0%).

### Stride or step length

**Figure 5:**
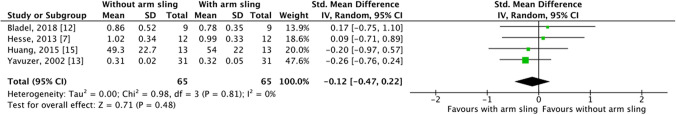
Forest plot for step or stride length comparison.

Four studies examined the stride or step length of patients using and not using an arm sling^7, 12, 13, 15^. As Fig. 5 shows, no significant between-group differences were detected (*P* = 0.48). However, an evident trend favoring the arm sling–using group was observed (SMD =  − 0.12, 95% CI − 0.47 to 0.22, n = 65). The results indicated low heterogeneity among the studies (*I*^2^ = 0%).

### Cadence

**Figure 6:**
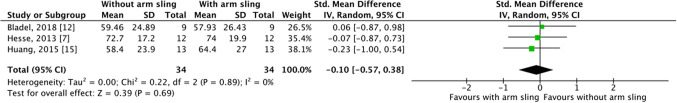
Forest plot for cadence comparison.

Three studies examined the cadence of patients using and not using an arm sling^7, 12, 15^. As Fig. 6 shows, no significant between-group differences were found (*P* = 0.69). However, an evident trend favoring arm sling–using group was noted (SMD =  − 0.1, 95% CI − 0.57 to 0.38, n = 34). The results indicated low heterogeneity among the studies (*I*^2^ = 0%).

No adverse effect was reported in any of the nine trials. However, the studies by Sohn et al. and Han et al. mentioned concerns over the increased flexor synergy pattern and interference of functional activities during arm sling use^1, 10, 16^.

## Discussion

The result of our study illustrated that arm sling use improved gait by significantly increasing walking speed in patients with post-stroke hemiplegia. Multiple studies have demonstrated walking speed to be critically associated with gait performance, quality of life, social participation, and even the ability to return to employment^19–22^.

Moreover, previous studies showed hemiplegic patients demonstrated gait abnormality including decreased cadence and stride/step length^23, 24^. Hwang et al. evaluated both step and stride length for the hemiplegic side and non-hemiplegic side of patients with stroke. We selected stride length on the hemiplegic side for further analysis due to its greatest clinical significance^15, 25^. Step and stride length showed trends favoring the arm sling–using groups in the included studies, but these differences were not significant, indicating that the improvement in walking speed we noted may be associated with changes in multiple parameters.

Numerous theories have been proposed for the biomechanical changes of arm sling application, including an inhibition of abnormal arm–trunk movement patterns and a decrease in the excursion of the center of gravity, both of which are frequently observed in patients with hemiplegia. Furthermore, these studies also mentioned that arm slings may help patients become more aware of their upper limbs and body posture, which in turn makes them felt more secure and facilitates postural adaptations^7, 13, 14^. Two studies reported that following stroke, patients experienced increased energy cost and poorer energy efficiency when walking^26, 27^. Among our included studies, both Jeong et al. and Han et al. reported more efficient walking when using an arm sling, with a reduced oxygen cost per meter observed^10, 17^.

Although no significant between-group difference was detected in balance performance, we noted a trend favoring the arm sling–using group. The Berg Balance Scale was selected over other assessment tools not only because of our study design but also because of its well-established application and clinical importance^28, 29^. Meanwhile, the Kinesthetic Ability Trainer is an emerging technique for balance evaluation. One study reported it as a reliable test for both static and dynamic balance and it displayed a moderate-to-high correlation with the Berg Balance Scale^30^.

Some differences between the types of arm slings used in the included studies were observed. Sohn et al. applied two types of slings, Bobath slings and simple arm slings^16^. We selected the simple arm sling for further analysis due to its higher similarity to the sling types used in other studies. Anke et al. also used two types of slings, Actimove and Shoulderlift, but only very small differences were noted between their effects on walking speed and stride length^12^. We selected Actimove for further analysis because it displayed marginally better improvement compared with Shoulderlift. Studies have demonstrated that different types of slings have diverse effects on shoulder subluxation. No one superior type has been identified; rather, because of the difference in mechanisms, individualized selection was recommended^8, 31^.

In quality assessment, we observed that three of the nine included studies did not describe the randomization. Concealed allocation was not applied in any of the studies. However, the crossover design may have reduced the degree of bias. Blinding was not achieved in any of the included studies, as expected, because of the characteristics of arm slings as orthoses. Blinding in such studies is difficult if not impossible. Considering these factors, all of our studies were of medium quality on the PEDro scale, which was sufficient for comparison trials evaluating arm sling application.

One review article, Anke et al., was published recently, which had reported minor effects on balance or gait when wearing an arm sling. However, it did not carry out meta-analysis^32^. Due to the absence of of quantitative analysis, the significance of results could not be revealed in this study. On the contrary, due to the significant result and the trends noticed in our study, we considered this topic a field worthy for further investigation, while large-scale clinical trial remained lacking currently.

One concern has to be mentioned was the short intervention periods noticed in nearly all the studies. Most of the studies let the patients wear on arm slings briefly before test. This disadvantage was also mentioned by Anke et al.^32^ Only one study, Hesse et al., applied the arm sling one week prior to the outcome measurement^7^. Hence, the fact that finding of our study was one with short-term intervention should be specified. Meanwhile, the lack of data on the long-term intervention might also cause the uncertainty toward the report of adverse effect.

Our study has several strengths. First, low heterogeneity was observed among the studies. Second, all the studies used crossover designs, which reduced the effect of confounding covariates. Third, this was the first meta-analysis of the effect of arm slings on gait and balance.

Our study had several limitations. First, blinding was not achieved in the included studies, and the outcomes were subjective, which may have lowered the reliability of the results. Second, the crossover designs used by all the included studies may result in the carryover effect if the length of the washout period was insufficient. However, because arm slings are orthoses, their application may not have carryover effects substantial enough to interfere with the true treatment effect. Third, most of the included studies had small sample sizes.

In conclusion, arm sling use was an effective and appropriate clinical intervention for patients with post-stroke hemiplegia that improved gait performance especially walking speed. Arm slings may help prevent abnormal gait patterns and lead to increases in sense of security, awareness of the hemiplegic side, and walking energy efficiency. Future studies should focus on the establishment of protocols for more specific and individualized arm sling application.
